# Conformal SiNx Coating on Carbon Nanotubes via Transient UV–Ozone Functionalization and Two-Step Atomic Layer Deposition

**DOI:** 10.3390/ma19101919

**Published:** 2026-05-07

**Authors:** Young Woo Kang, Haneul Kim, Inseo Lee, Yongkyung Kim, In-Sung Park, Jinho Ahn

**Affiliations:** 1 Division of Materials Science and Engineering, Hanyang University, Seoul 04763, Republic of Korea; kyw9412@naver.com (Y.W.K.); milky-way@nate.com (H.K.); inseo000616@gmail.com (I.L.); kek1144@hanyang.ac.kr (Y.K.); 2 CHIPS Innovation Research Center, Hanyang University, Seoul 04763, Republic of Korea; 3 Institute of Nano Science and Technology, Hanyang University, Seoul 04763, Republic of Korea

**Keywords:** carbon nanotubes, atomic layer deposition, surface functionalization, conformal coating, UV–ozone treatment

## Abstract

A conformal SiNx coating on carbon nanotubes (CNTs) was achieved by combining transient UV–ozone surface functionalization with a two-step atomic layer deposition (ALD) process. UV–ozone treatment gradually increased the defect density of CNTs, with the I_D_/I_G_ ratio increasing from 0.05 for pristine CNTs to 0.25 after 7 min of exposure, while the overall fibrous CNT network remained intact. However, prolonged UV–ozone exposure beyond 10 min led to a sharp increase in the I_D_/I_G_ ratio to 0.46, accompanied by structural degradation of the CNT membrane. Hydroxyl (-OH), epoxy (C-O-C), and carbonyl (C=O) groups were introduced by UV–ozone treatment and were partially removed during subsequent high-temperature processing. Accordingly, direct high-temperature ALD resulted in incomplete SiNx coverage of the CNTs, suggesting insufficient nucleation. A two-step ALD process, consisting of several cycles of low-temperature nucleation at 100 °C followed by high-temperature growth at 700 °C, enabled more conformal deposition of SiNx on CNTs. In addition, both annealing and ALD reduced the defect level toward that of pristine CNTs, supporting the transient nature of UV–ozone-induced functionalization.

## 1. Introduction

Carbon nanotubes (CNTs) have attracted considerable attention as one-dimensional carbon nanostructures because of their exceptional mechanical strength, high electrical and thermal conductivities, and low density [1,2]. CNTs are used in a wide range of applications including separation membranes, chemical sensors, electrochemical devices, and optical filters [3,4,5,6]. In these systems, thin conformal coatings are often introduced to improve the chemical and thermal stability, protect the CNT framework in harsh operating environments, and impart application-specific properties. Among these applications, CNT-based extreme ultraviolet (EUV) pellicles provide a representative example in which such coatings are required. Although CNT pellicles offer high optical transmittance and mechanical robustness, they are chemically vulnerable in EUV scanner environments where EUV-induced hydrogen plasma can progressively etch and degrade the CNT structure [7,8]. Accordingly, ultrathin capping layers have been proposed as a strategy to suppress hydrogen-induced degradation and extend pellicle lifetime [9,10]. Thus, conformal coating of the porous CNT membrane is essential to ensure that individual CNT filaments can be uniformly protected without sacrificing the optical and structural advantages of the membrane.

However, the uniform deposition of materials onto CNTs remains challenging. Unlike planar substrates, CNTs exhibit a highly complex three-dimensional morphology, and the deposited layer must conformally wrap the individual CNT filaments without excessively increasing the effective thickness or obstructing the open network structure. These requirements are especially critical in applications involving ultrathin coatings, where incomplete surface coverage or excessive film growth can severely deteriorate device performance.

Atomic layer deposition (ALD) is regarded as one of the most promising techniques for this purpose because of its sequential, self-limiting surface reactions and excellent conformality on high-aspect-ratio nanostructures [11]. Nevertheless, pristine CNT surfaces exhibit intrinsically poor ALD nucleation behavior because their sp^2^-bonded graphitic surface provides only a limited number of reactive adsorption sites for ALD precursors and reactants [12,13]. Consequently, deposition on untreated CNTs often leads to delayed nucleation and discontinuous island growth rather than the formation of a uniform ultrathin coating.

Various surface-activation strategies have been explored to promote ALD nucleation on chemically inert graphene and CNT surfaces. A widely used approach involves plasma-based treatments or aggressive oxidation processes to generate reactive defect sites on the carbon surface [14]. Although these methods can significantly increase the nucleation density, they inevitably introduce structural damage or etching of the carbon framework, which can degrade the intrinsic mechanical integrity of the CNT networks.

Alternatively, nondestructive strategies based on molecular functionalization or polymer seed layers have been investigated. For example, the adsorption of aromatic molecules such as perylene tetracarboxylic acid and the use of polymer buffer layers facilitates uniform ALD growth on graphitic surfaces by providing functional groups for precursor adsorption [12]. However, these approaches inevitably introduce an additional interfacial layer between the CNT surface and the deposited film [15,16]. Such interlayers are undesirable for applications requiring ultrathin coatings because they increase the effective film thickness and can introduce optical absorption or scattering. In particular, for CNT pellicles, where minimal thickness and high optical transparency of the coating are essential, the presence of organic or polymeric layers can significantly compromise the optical performance. Therefore, a surface-modification strategy that enhances ALD nucleation while minimizing structural damage and avoiding residual interfacial layers is required.

In this study, we propose UV–ozone-induced surface functionalization as a transient strategy to enable conformal atomic layer deposition (ALD) on carbon nanotubes (CNTs) for barrier coating applications. The oxygen-containing functional groups introduced by UV–ozone treatment serve as temporary adsorption sites for ALD precursors and reactants, thereby promoting initial nucleation and enabling uniform coating formation at nanometer-scale thicknesses. However, because these functional groups are partially desorbed during subsequent high-temperature processing, direct high-temperature ALD alone cannot maintain sufficient nucleation sites for continuous ultrathin coating growth. To overcome this limitation, we introduce a two-step ALD process consisting of a low-temperature nucleation step and a high-temperature growth step. In this approach, the low-temperature step preserves the transient functional groups and maximizes initial SiNx nucleation on the CNT surface, while the subsequent high-temperature step enables continued film growth on the pre-formed nuclei even after partial desorption of the functional groups. Through combined spectroscopic and microscopic analyses, we clarify how transient surface functionalization and temperature-separated ALD processing govern nucleation behavior and conformal coating formation on chemically inert CNT surfaces. In this sense, developing conformal ultrathin coating methods addresses the key challenge of balancing protective coverage with preservation of nanoscale material characteristics.

## 2. Materials and Methods

### 2.1. UV–Ozone Surface Functionalization of CNT Membranes

The fabrication of the freestanding CNT membranes and their physical and chemical properties were reported already [17]. The membranes were derived from CNT aerogels synthesized by floating catalyst chemical vapor deposition (FCCVD) using ferrocene and thiophene as the catalyst precursor and growth promoter, respectively, with CH_4_, H_2_, and Ar as the process gases. The as-grown CNT aerogel was continuously collected by direct winding to form a self-supporting CNT film and subsequently transferred onto a silicon support frame with a 10 mm × 10 mm aperture. Representative characterization in the supplier group’s report indicated predominantly double-walled CNTs with few-nanometer diameters. These values are provided as representative background information, because the exact batch used in this study was supplied externally and was not fully re-characterized in our laboratory. Prior to surface functionalization, the CNT pellicles were thermally treated at 400 °C for 30 min under a low vacuum (~1 × 10^−4^ Pa) to remove adventitious contaminants and weakly adsorbed surface species introduced during handling in ambient conditions.

Surface functionalization was performed using a UV–ozone cleaner under ambient atmospheric conditions. The system employed 254 nm and 183 nm UV lamps, and the membranes were placed approximately 10 cm from the UV source. No additional gas flow or active temperature control was used, and the treatment was therefore carried out in ambient air near room temperature. The ozone concentration was not independently measured. The membranes were exposed to UV-generated ozone for controlled durations of up to 15 min to induce mild oxidation of the CNT surface. The purpose of this treatment was not to generate permanent structural defects, but to introduce temporary oxygen-containing functional groups (e.g., hydroxyl and ether species) that could act as adsorption sites for the ALD precursors and reactants. The exposure time was carefully controlled to balance the functional group formation and structural preservation of the CNT network. To evaluate the reversibility of the surface modification, selected samples were subjected to post-treatment annealing at 700 °C under an inert atmosphere. This temperature was selected to reproduce the high-temperature growth condition of the subsequent SiNx ALD process, thereby allowing the thermal desorption of UV–ozone-induced functional groups and the associated structural restoration of CNTs to be examined independently from ALD precursor reactions.

### 2.2. Thermal ALD of SiNx

**Figure 1:**
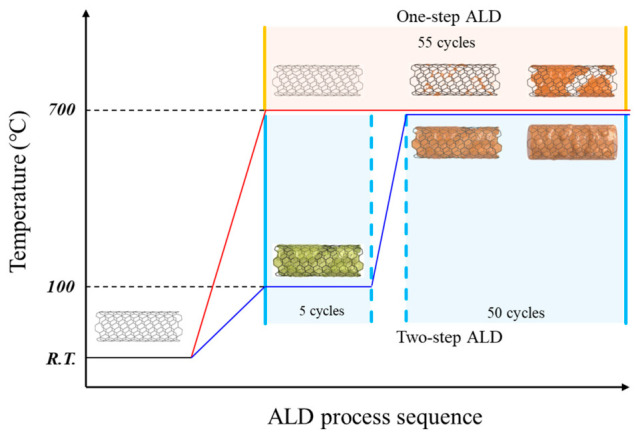
Schematic comparison of the one- and two-step SiNx ALD processes on CNTs.

SiNx films were deposited onto pristine and UV–ozone-functionalized CNT membranes using homemade tube furnace type thermal ALD [18]. Si_2_Cl_6_ and NH_3_ were used as the Si precursor and N reactant, respectively, and N_2_ was used as the carrier and purge gas. Based on the previously established high-temperature SiNx ALD condition, one ALD cycle consisted of a Si_2_Cl_6_ pulse (1 s), N_2_ purge (20 s), NH_3_ pulse (2 s), and N_2_ purge (20 s). The deposition was performed primarily to evaluate the influence of surface functionalization on the coverage and conformality of the ultrathin coatings on individual CNT filaments. As illustrated in Figure 1, two different deposition approaches were employed to compare the growth behaviors of SiNx on the CNT surfaces.

In the one-step ALD process, the entire deposition was performed directly at 700 °C, corresponding to 55 cycles under the conventional deposition condition. This process was used to evaluate the nucleation behavior of pristine and UV–ozone-treated CNTs without an additional low-temperature seed step.

To more effectively use the oxygen-containing functional groups introduced by the UV–ozone treatment as adsorption sites for the ALD precursors, a two-step ALD process was additionally implemented. In the first step, a low-temperature deposition was carried out at 100 °C for 5 cycles to promote precursor adsorption and maximize initial nucleation on the functionalized CNT surface. As shown in Figure 1, this step was intended to preserve the thermally unstable surface functional groups introduced by UV–ozone treatment and to facilitate initial nucleation on the CNT surface before the subsequent high-temperature growth step. In the second step, the substrate temperature was increased to 700 °C for continued deposition over 50 cycles, resulting in a final film thickness of approximately 5 nm. This temperature-escalation process enabled subsequent film growth from the nuclei formed during the initial low-temperature step, thereby improving coating continuity and conformality.

### 2.3. Chemical and Structural Characterization

Raman spectroscopy was performed to evaluate the structural disorder and defect evolution of the CNTs after UV–ozone treatment and subsequent thermal processing. The spectra were acquired using a LabRAM Aramis Raman microscope (Horiba, Kyoto, Japan) with a 514 nm excitation laser, a 50× objective, a 600 gr/mm grating, and a spot size of approximately 1 μm. The laser power on the sample was kept below 1 mW to minimize local heating and damage to the CNT membranes. The ratio of the D-band to the G-band intensity (I_D_/I_G_) was used to quantify the changes in the defect density.

X-ray photoelectron spectroscopy (XPS) was performed using a Thermo Fisher Scientific Nexsa system with an Al Kα X-ray source and a 400 μm spot size. Survey spectra were acquired in CAE mode with a pass energy of 200 eV, an energy step size of 1.0 eV, and 3 scans. Narrow scan spectra were acquired in CAE mode with a pass energy of 50 eV and an energy step size of 0.1 eV; the C 1s spectra were collected using 10 scans and the O 1s spectra using 25 scans. All binding energies were calibrated to the graphitic sp^2^ C=C peak at 284.5 eV. Peak fitting was performed using XPSPEAK41 with a Shirley background. XPS was used in this study primarily for qualitative identification of oxygen-containing functional groups on the CNT surface and for comparative analysis of their relative changes after UV–ozone treatment and subsequent thermal processing.

Scanning electron microscopy (SEM) was employed to examine the morphological integrity of the CNT membrane after UV–ozone treatment, allowing the assessment of potential structural damage, such as filament breakage or etching, at the network scale. Transmission electron microscopy (TEM) was used to directly observe the morphology and conformality of the SiNx coatings on individual CNT filaments, enabling a comparison between island-type nucleation and continuous conformal coating at ultrathin thicknesses.

## 3. Results and Discussion

### 3.1. UV–Ozone-Induced Functionalization of CNTs

**Figure 2:**
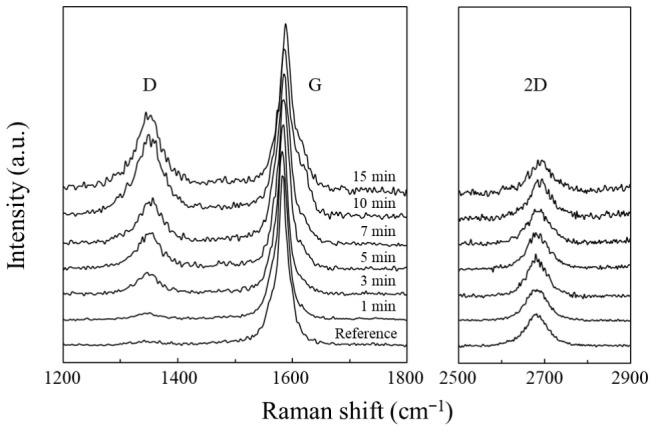
Raman spectra of the CNT membranes as a function of the UV–ozone exposure time. The evolution of the D, G, and 2D bands indicates progressive defect formation while preserving the overall graphitic structure.

UV–ozone treatment was employed to introduce oxygen-containing functional groups onto the CNT surface, and the resulting structural modifications were analyzed using Raman spectroscopy. Figure 2 shows the Raman spectra of the CNT membranes as a function of the UV–ozone exposure time. The characteristic D (~1350 cm^−1^), G (~1580–1600 cm^−1^), and 2D (~2700 cm^−1^) bands corresponding to graphitic carbon were clearly observed for all samples. Although the overall spectral features of the CNTs were preserved after UV–ozone exposure, noticeable changes in the relative peak intensities were observed with increasing treatment time.

In particular, the intensity of the D band gradually increased as the UV–ozone exposure time increased. The D band originates from the intervalley double-resonance scattering associated with structural defects or sp^3^-hybridized carbon sites in graphitic materials. Therefore, the increase in D-band intensity indicates the introduction of defect sites within the CNT lattice. In the case of UV–ozone treatment, these defects are primarily attributed to the formation of oxygen-containing functional groups, such as hydroxyl, carbonyl, or epoxy species, which locally convert sp^2^-hybridized carbon into sp^3^ configurations [19,20]. In addition, a slight shift in the G band was observed after UV–ozone exposure. Such peak shifts have been reported in oxidatively functionalized CNTs and are generally attributed to charge-transfer interactions between the π-electron system of CNTs and oxygen-containing functional groups. The resulting modification of the electronic structure can lead to a detectable shift in the G-band position, suggesting a doping-like effect induced by surface functionalization.

**Figure 3:**
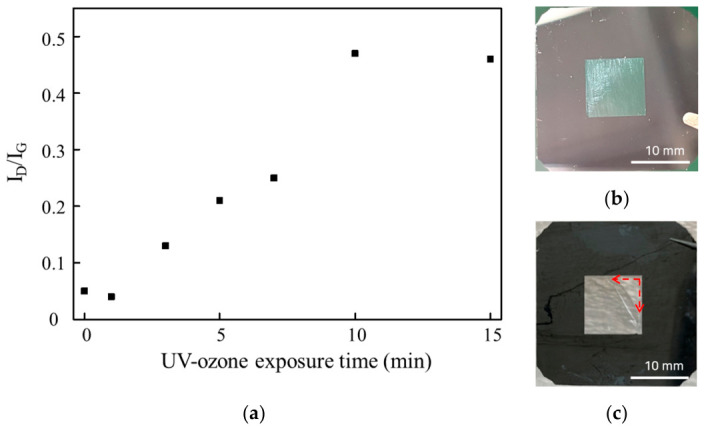
(**a**) Evolution of the I_D_/I_G_ ratio of CNT membranes as a function of the UV–ozone exposure time. (**b**) Pristine CNT membrane. (**c**) CNT membrane after 10 min of UV–ozone exposure. The image comparison shows that excessive UV–ozone treatment leads to mechanical failure of the membrane.

To quantitatively evaluate the degree of structural modification, the ratio of the D-band to the G-band intensity (I_D_/I_G_) was extracted from the Raman spectra and plotted as a function of the UV–ozone exposure time, as shown in Figure 3a The I_D_/I_G_ ratio progressively increased with increasing exposure time, from 0.05 for pristine CNTs to 0.25 after 7 min of UV–ozone exposure, indicating a gradual increase in defect density within the CNT structure. However, prolonged exposure beyond 10 min led to a sharp increase in the I_D_/I_G_ ratio to 0.46, suggesting an excessive disruption of the sp^2^ carbon network. This increase is attributed to the formation of oxygen-containing functional groups and the associated introduction of sp^3^-like carbon configurations, which alter the intrinsic bonding structure of CNTs. Although sp^3^ bonding is generally stronger at the atomic level, its incorporation into a conjugated sp^2^ carbon network reduces the flexibility of CNT filaments and increases their brittleness [14,21].

The mechanical consequences of excessive functionalization are evidenced by the optical images in Figure 3b,c. While the pristine CNT membrane maintained an intact square membrane region without visible mechanical damage (Figure 3b), the CNT membrane exposed to UV–ozone for 10 min exhibited tearing that initiated from the membrane edge (Figure 3c). Although the mechanical stress state of the membrane was not directly quantified in the present study, the edge-initiated failure suggests that prolonged UV–ozone exposure increases the susceptibility of the membrane to mechanical damage. Combined with the Raman results showing substantial defect accumulation, this observation indicates that excessive functionalization compromises the structural integrity of the CNT membrane. These results indicate that prolonged UV–ozone exposure not only modifies the surface chemistry of CNTs but also compromises the structural stability of the CNT membrane.

**Figure 4:**
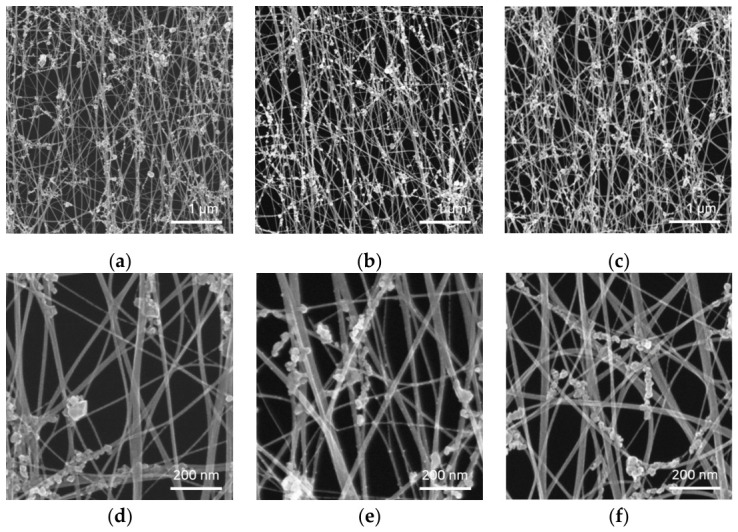
SEM images of CNT membranes under different UV–ozone exposure conditions: (**a**,**d**) pristine CNT, (**b**,**e**) 7 min of exposure, and (**c**,**f**) 10 min of exposure. Low-magnification images are shown in (**a**–**c**), and high-magnification images are shown in (**d**–**f**). The fibrous CNT network was largely maintained after UV–ozone exposure, without noticeable filament-scale etching or loss of CNT strands. The particle-like features along the CNT filaments are attributed to residual catalyst particles from CNT growth.

To further investigate whether the observed membrane failure originated from the direct structural destruction of the CNT filaments, SEM analysis was performed (Figure 4). The SEM images of the pristine CNT membranes and those exposed to UV–ozone for 7 and 10 min revealed that the fibrous CNT network remained largely intact, with no clear evidence of severe etching, filament breakage, or disappearance of CNT strands at the microscale. The overall network morphology, including the interconnected filament structure, was preserved even under exposure conditions where macroscopic tearing occurred.

These observations suggest that UV–ozone treatment does not induce catastrophic removal of CNT filaments but rather modifies the surface chemistry through the introduction of oxygen-containing functional groups. Combined with the Raman analysis, the results indicate that prolonged UV–ozone exposure leads to the accumulation of surface defects and sp^3^-like bonding, which reduces the mechanical robustness of individual CNT filaments and the overall network. Consequently, membrane failure is more likely to be associated with defect-induced degradation of the mechanical integrity rather than direct etching of the CNT structure.

Based on these results, an optimized UV–ozone exposure window is required to achieve sufficient surface functionalization for subsequent ALD nucleation and the preservation of structural integrity. Exposure times shorter than 7 min provide an effective compromise, enabling controlled functionalization while avoiding significant mechanical degradation of the CNT membranes.

### 3.2. Chemical Analysis of UV–Ozone Functionalized CNT Surfaces

**Figure 5:**
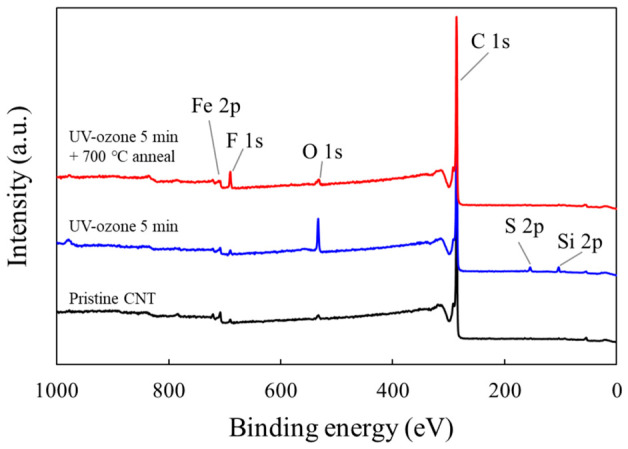
XPS spectra of pristine CNT, 5 min UV–ozone exposed CNT, and 700 °C annealed CNT after 5 min UV–ozone exposure analyzed at the membrane region.

The chemical modification of the CNT surfaces induced by the UV–ozone treatment was investigated using XPS. Figure 5 shows the survey spectra of pristine CNTs, UV–ozone-treated CNTs (5 min), and UV–ozone-treated CNTs followed by thermal annealing at 700 °C. The pristine CNT sample exhibited a dominant C 1s peak with negligible oxygen-related signals, indicating the inherently inert nature of the CNT surface. After UV–ozone exposure, a clear increase in the O 1s signal was observed, confirming the introduction of oxygen-containing species onto the CNT surface. This trend was also reflected in the survey spectra, where the oxygen fraction increased from 1.6 at.% for pristine CNTs to 7.6 at.% after 5 min UV–ozone exposure, and then decreased to 2.5 at.% after subsequent annealing at 700 °C, while the carbon fraction changed in the opposite direction. Notably, the O 1s intensity decreased after high-temperature annealing, suggesting the partial removal of oxygen functional groups and indicating the reversible nature of the UV–ozone-induced surface modification. Weak additional signals were also observed in the survey spectra, including Fe-related features attributed to residual catalyst species and trace F-, S-, and Si-related signals regarded as minor impurity/background contributions.

**Figure 6:**
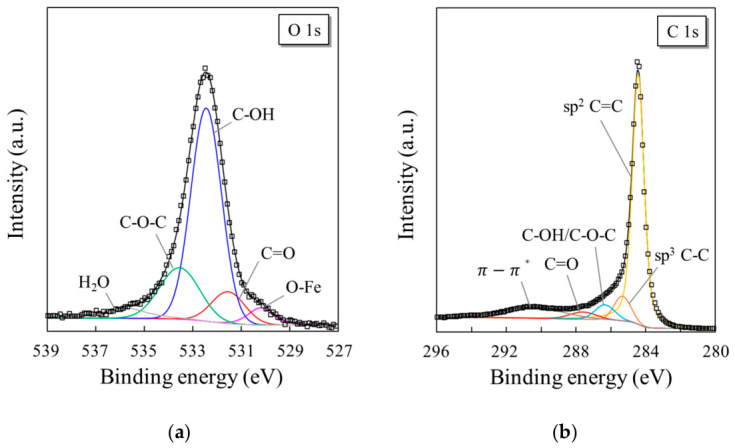
Deconvolution of the (**a**) O 1s and (**b**) C 1s peaks in the XPS narrow spectra obtained from the CNT membrane after 5 min of UV–ozone exposure.

To further analyze the chemical states of the introduced oxygen species, the O 1s core-level spectrum of the UV–ozone-treated CNTs was deconvoluted, as shown in Figure 6a. The O 1s peak can be fitted to multiple components corresponding to different oxygen functional groups. The dominant peak located at ~532–533 eV was attributed to hydroxyl (C–OH) groups, whereas the component at ~533–534 eV corresponded to epoxy (C–O–C) bonds. A smaller contribution at a lower binding energy (~531–532 eV) was assigned to carbonyl (C=O) species. To account for the shoulder on the lower binding energy side of the O 1s spectrum, an additional component near ~530–531 eV was introduced and attributed to oxygen bound to residual metal catalyst species, most plausibly Fe–O. At the higher binding energy side, a weak peak at a higher binding energy (~535–536 eV) was associated with adsorbed H_2_O or loosely bound oxygen species on the CNT surface. These peak assignments are consistent with those of the previously reported XPS analyses of oxidized carbon nanotubes [22].

Consistent with the O 1s analysis, the C 1s spectrum (Figure 6b) also exhibited clear signatures of oxygen functionalization. The main peak at ~284.5 eV corresponded to sp^2^-hybridized C=C bonds in the graphitic CNT structure, while a component in the ~285.0–285.5 eV region was attributed to sp^3^-related carbon (C–C/C–H) arising from structural disorder and/or oxygen functionalization. Higher binding energy components were assigned to oxygen-containing groups, including C–O and C=O species, together with a weak π–π* shake-up feature. The peak at ~285.5–286.0 eV was attributed to C–O bonds (including C–OH and C–O–C species), whereas the peak at ~287–288 eV corresponded to the C=O groups. A weak feature at ~290–291 eV was assigned to π–π* shake-up transitions, characteristic of conjugated graphitic systems.

The presence of C–O and C=O components in the C 1s spectrum is in good agreement with the functional groups identified in the O 1s spectrum, confirming that the UV–ozone treatment introduced oxygen-containing species onto the CNT surface in a chemically consistent manner. Notably, the persistence of the strong sp^2^ C=C peak and the π–π* feature indicates that the overall graphitic structure was largely maintained, suggesting that the oxidation was limited to surface functionalization rather than bulk structural degradation.

### 3.3. Effect of UV–Ozone Functionalization on ALD Coating Behavior and Structural Recovery

**Figure 7:**
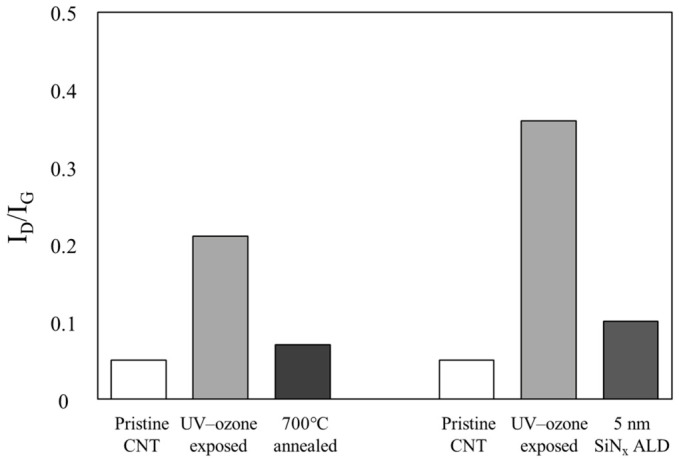
Comparison of the I_D_/I_G_ ratio of pristine CNTs, UV–ozone-treated CNTs, and post-processed CNTs after 700 °C thermal annealing or 5 nm SiNx ALD. Both post-treatments reduced the defect ratio through removal of oxygen-related defects, indicating partial restoration of the CNT structure after transient UV–ozone functionalization.

In the present two-step process, 700 °C was used as the second-step growth temperature in the context of thermal SiNx ALD, where elevated temperatures are often required to achieve sufficient NH_3_ reactivity and nitride formation. Because such a temperature can also independently influence CNT surface chemistry, a separate 700 °C annealing control was included to distinguish thermal removal of UV–ozone-induced oxygen species from changes associated with precursor-assisted growth. To further investigate the evolution of CNT structural integrity after SiNx deposition, the defect ratio (I_D_/I_G_) was analyzed before and after the ALD process (Figure 7). As discussed in Section 3.1, UV–ozone treatment significantly increased the defect density of the CNTs after 5 min of exposure. However, after subsequent SiNx deposition, the I_D_/I_G_ ratio markedly decreased, approaching that of pristine CNT. This reduction indicates that a large fraction of the defects introduced by UV–ozone treatment were associated with oxygen-containing functional groups, which were removed during the high-temperature ALD process, consistent with their thermal instability [23,24]. At the same time, the I_D_/I_G_ ratio did not fully recover to the pristine level, suggesting that UV–ozone treatment may also induce a small amount of irreversible structural defects. Taken together with the SEM observation that the fibrous CNT network remained largely intact after UV–ozone exposure and the XPS evidence that oxygen-containing species were reduced after subsequent high-temperature processing, these results suggest that a substantial fraction of the UV–ozone-induced disorder is associated with removable oxygen functionalities rather than extensive irreversible disruption of the CNT framework. In this sense, the UV–ozone treatment can be regarded as a transient surface activation step, because its primary effect is to temporarily introduce adsorption sites for ALD nucleation while allowing partial restoration toward the original CNT framework after high-temperature processing.

**Figure 8:**
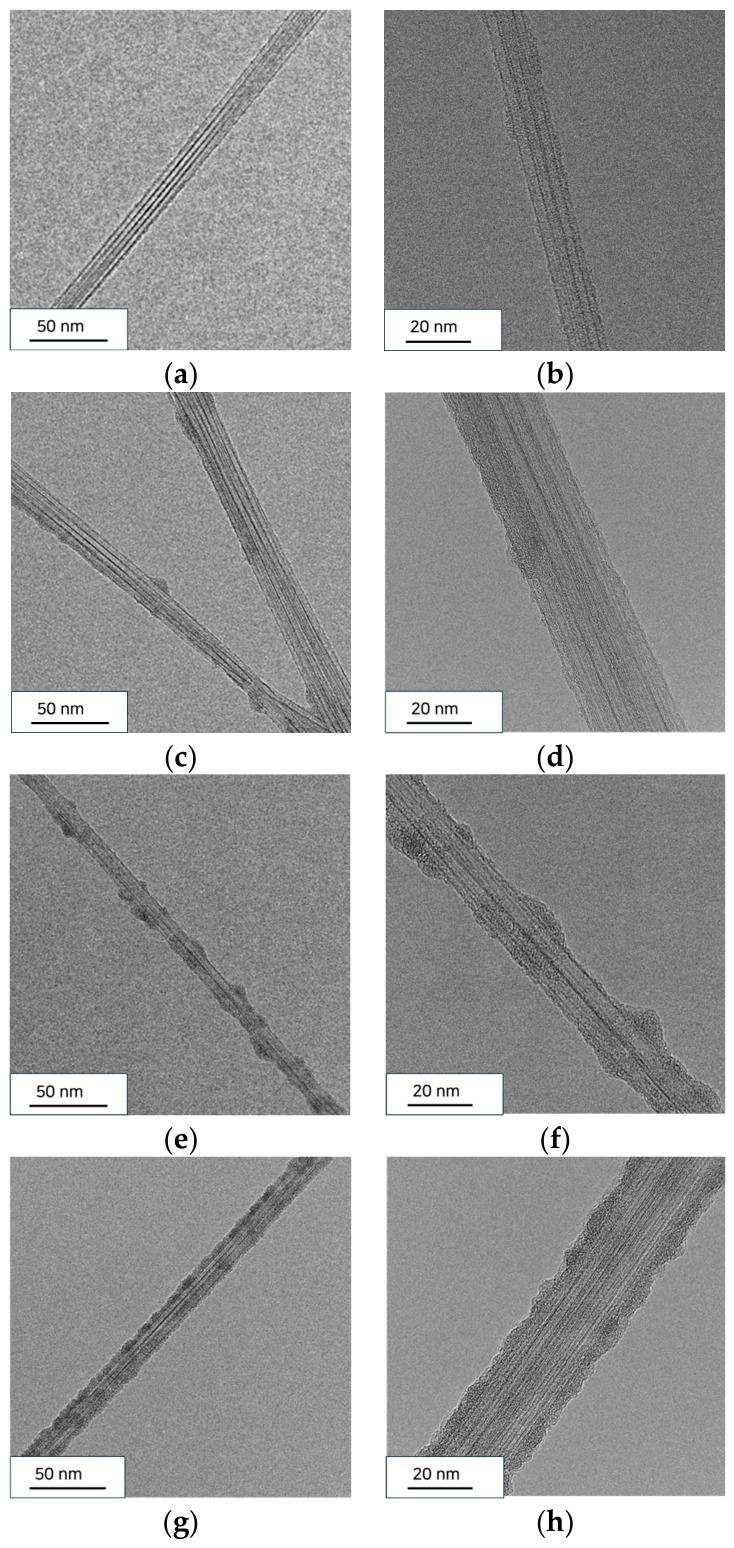
TEM images showing the effect of UV–ozone functionalization and ALD process conditions on the SiNx coating morphology on CNTs: (**a**,**b**) pristine CNT, (**c**,**d**) SiNx deposited on pristine CNT, (**e**,**f**) SiNx deposited on UV–ozone-treated CNT (5 min of exposure) by the one-step ALD process, and (**g**,**h**) SiNx deposited on UV–ozone-treated CNT by the two-step ALD process (low-temperature nucleation followed by high-temperature growth). Panels (**a**,**c**,**e**,**g**) show lower-magnification images, and panels (**b**,**d**,**f**,**h**) show higher-magnification images.

Because the deposited SiNx layer was ultrathin (~5 nm), its direct contribution to the Raman spectrum is expected to be limited. In particular, SiNx does not typically exhibit distinct Raman features in the spectral region of the CNT D and G bands, and therefore significant peak overlap is not expected. Nevertheless, the presence of the coating may still affect the absolute Raman intensity through attenuation or other optical effects. Accordingly, the post-ALD Raman results are interpreted in a comparative and semiquantitative manner, and the decrease in I_D_/I_G_ is taken as being consistent with partial removal of oxidation-induced surface species during high-temperature processing rather than as direct proof of complete structural restoration of the pristine CNT framework. The effect of this activated surface on the subsequent SiNx coating morphology was further examined by TEM analysis (Figure 8).

The effect of this transient activation strategy on the SiNx coating morphology was further examined using TEM analysis (Figure 8). Figure 8a,b shows the pristine CNTs without coatings, which exhibited a clean and smooth graphitic surface with no observable amorphous layers. When SiNx was deposited directly on the pristine CNTs (Figure 8c,d), the coating appeared discontinuous and nonuniform, with isolated SiNx clusters formed along the CNT surface. This island-like morphology indicates that the inert sp^2^ carbon surface provides insufficient nucleation sites, resulting in poor surface coverage, even at a target thickness of ~5 nm. Such nonconformal growth is characteristic of ALD on chemically inert carbon materials, where limited precursor adsorption leads to delayed nucleation.

By contrast, the UV–ozone-treated CNTs exhibited a significantly improved coating behavior. As shown in Figure 8e,f, SiNx deposited on CNTs exposed to UV–ozone for 5 min by the one-step ALD process showed an increased nucleation density and more uniformly distributed SiNx islands compared to the pristine case. This result indicates that the oxygen-containing functional groups introduced by the UV–ozone treatment effectively enhanced the initial adsorption of the ALD precursors. However, the film still contained locally nonuniform regions and incomplete coverage, suggesting that nucleation enhancement alone was not sufficient to achieve a fully conformal ultrathin coating. This incomplete coverage was likely associated with the partial loss of surface functional groups under the high-temperature ALD conditions, which reduced the number of active sites available for sustained growth [25].

To overcome this limitation, a two-step ALD process, consisting of a low-temperature nucleation step followed by high-temperature growth, was employed. As shown in Figure 8g,h, this approach resulted in a highly uniform and conformal SiNx layer that continuously wrapped the individual CNT filaments. The coating exhibited a consistent thickness and smooth morphology along the CNT surface, indicating that the combination of UV–ozone functionalization and the optimized ALD sequence effectively promoted uniform precursor adsorption and continuous film formation. The chemical identity of this conformal ultrathin layer is further supported by our previous analysis of Si_2_Cl_6_/NH_3_ thermal ALD SiNx deposited under the same reactor conditions, where XPS confirmed dominant Si–N bonding with only a minor Si–O contribution (<8%), consistent with silicon nitride coatings [18]. Together with the continuous shell morphology observed here, this supports that the ultrathin layer conformally wrapping the CNT filaments is SiNx.

These results demonstrate that UV-ozone-induced functional groups serve as temporary nucleation sites for ALD, and that their transient nature can be advantageously combined with a two-step deposition strategy. Consequently, ultrathin SiNx coatings with improved continuity and conformality can be achieved on CNTs while preserving the intrinsic structure of the underlying carbon framework. These outcomes are directly relevant to the research motivation of realizing ultrathin protective coatings, since coating continuity, minimal thickness increase, and preservation of the CNT network represent key performance-related requirements for such applications.

## 4. Conclusions

Transient UV–ozone treatment provided a controllable means of temporarily activating the chemically inert CNT surface for SiNx deposition while preserving the overall fibrous network within an optimized treatment window. The defect level gradually increased with UV–ozone exposure and remained within a tolerable range for up to 7 min, whereas prolonged treatment caused a sharp increase in structural disorder and led to the degradation of the CNT membrane. Oxygen-containing surface functionalities introduced by UV–ozone treatment promoted initial nucleation, but their partial loss during subsequent high-temperature processing resulted in incomplete coverage during direct high-temperature ALD. By contrast, a two-step ALD process combining low-temperature nucleation with high-temperature growth enabled the formation of a conformal 5 nm SiNx coating on individual CNT filaments. The subsequent decrease in the I_D_/I_G_ ratio after annealing or ALD was consistent with the removal of a substantial fraction of oxidation-induced surface species during high-temperature processing, indicating that the UV–ozone treatment acts predominantly as a transient surface activation step. Although a small fraction of irreversible defects likely remains, the results suggest partial restoration toward the original CNT framework rather than extensive permanent disruption.

Overall, this study establishes a practical and potentially scalable route for conformal coating of inert carbon nanostructures and provides mechanistic insight into how transient surface activation can be coupled with temperature-engineered ALD to achieve ultrathin continuous coatings. Importantly, the demonstrated enhancement in coating continuity and preservation of the CNT framework represents performance-enhancing outcomes directly related to the original motivation of achieving protective ultrathin coatings without compromising the intrinsic advantages of CNT membranes. Although this work focuses on the process-enabling and mechanistic aspects of conformal coating formation, the resulting approach provides a basis for future evaluation of application-level protective performance in systems such as CNT-based EUV pellicles, where both barrier functionality and structural preservation are critical.

## Data Availability

The original contributions presented in this study are included in the article. Further inquiries can be directed to the corresponding authors.

## Abbreviations

The following abbreviations are used in this manuscript:

CNT	Carbon nanotube
ALD	Atomic layer deposition
EUV	Extreme ultraviolet
XPS	X-ray photoelectron spectroscopy
SEM	Scanning electron microscopy
TEM	Transmission electron microscopy
